# A Mutation in the *Mesorhizobium loti oatB* Gene Alters the Physicochemical Properties of the Bacterial Cell Wall and Reduces Survival inside *Acanthamoeba castellanii*

**DOI:** 10.3390/ijms19113510

**Published:** 2018-11-08

**Authors:** Magdalena Anna Karaś, Anna Turska-Szewczuk, Małgorzata Marczak, Magdalena Jaszek, Monika Janczarek, Katarzyna Dworaczek, Dawid Stefaniuk, Jerzy Wydrych

**Affiliations:** 1 Department of Genetics and Microbiology, Institute of Microbiology and Biotechnology, Faculty of Biology and Biotechnology, Maria Curie-Sklodowska University, Akademicka 19 St., 20-033 Lublin, Poland; aturska@hektor.umcs.lublin.pl (A.T.-S.); malgorzata.marczak@poczta.umcs.lublin.pl (M.M.); mon.jan@poczta.umcs.lublin.pl (M.J.); pakiet.kat@gmail.com (K.D.); 2 Department of Biochemistry, Institute of Biology, Faculty of Biology and Biotechnology, Maria Curie-Sklodowska University, Akademicka 19 St., 20-033 Lublin, Poland; magdalena.jaszek@poczta.umcs.lublin.pl (M.J.); dawid.stefaniuk@umcs.pl (D.S.); 3 Department of Comparative Anatomy and Anthropology, Institute of Biology, Faculty of Biology and Biotechnology, Maria Curie-Sklodowska University, Akademicka 19 St., 20-033 Lublin, Poland; jerzy.wydrych@poczta.umcs.lublin.pl

**Keywords:** *Mesorhizobium loti*, *Acanthamoeba castellanii*, *O*-acetyltransferase, AFM, cell surface properties, rhizobia-amoebae interactions, lipopolysaccharide

## Abstract

In our previous report, we had shown that the free-living amoeba *Acanthamoeba castellanii* influenced the abundance, competiveness, and virulence of *Mesorhizobium loti* NZP2213, the microsymbiont of agriculturally important plants of the genus *Lotus*. The molecular basis of this phenomenon; however, had not been explored. In the present study, we demonstrated that *oatB*, the *O*-acetyltransferase encoding gene located in the lipopolysaccharide (LPS) synthesis cluster of *M. loti*, was responsible for maintaining the protective capacity of the bacterial cell envelope, necessary for the bacteria to fight environmental stress and survive inside amoeba cells. Using co-culture assays combined with fluorescence and electron microscopy, we showed that an *oatB* mutant, unlike the parental strain, was efficiently destroyed after rapid internalization by amoebae. Sensitivity and permeability studies of the *oatB* mutant, together with topography and nanomechanical investigations with the use of atomic force microscopy (AFM), indicated that the incomplete substitution of lipid A-core moieties with O-polysaccharide (O-PS) residues rendered the mutant more sensitive to hydrophobic compounds. Likewise, the truncated LPS moieties, rather than the lack of *O*-acetyl groups, made the *oatB* mutant susceptible to the bactericidal mechanisms (nitrosative stress and the action of lytic enzymes) of *A. castellanii*.

## 1. Introduction

When the rhizosphere is nitrogen-starved, legumes and the Gram-negative bacteria commonly called rhizobia enter into a facultative symbiosis, which enables the fixation of atmospheric dinitrogen. Rhizobia are also known as plant growth promoting bacteria (PGPR), and as such, they act as bio-fertilizers, phyto-stimulators, and rhizo-remediators for symbiotic and non-host plant species [1]. Thanks to those special features, they are used as bio-inoculants for sustainable agriculture. Rhizobium inoculants, however, must meet many requirements: they must be rhizospheric-competent and be able to colonize and survive in the rhizospheric soil, counteracting many fastidious factors present in it. Unfortunately, microorganisms that show promise in the lab may lack key characteristics for widespread adoption in sustainable and productive agricultural systems [2]. In particular, their survival capacity, and hence their impact on plant growth and health, may be limited by the influence of indigenous microflora, and especially protozoan grazing [3].

The species *Mesorhizobium loti* is a member of the rhizobia family of bacteria, which is able to form determinant-type globular nodules on several agriculturally important plants of the *Lotus* genus [4,5]. It has also been identified as a potential PGPR species with stimulating and protective activities [6]. *M. loti* NZP2213 (WT*—*wild type) is a representative strain that forms nitrogen-fixing nodules on *Lotus corniculatus*, and ineffective ones on *Lotus pedunculatus* and *Leucaena leucocephala*. The complete lipopolysaccharide (LPS) of the WT strain has been shown to be necessary for the establishment of effective symbiosis with the plant host [7]. Previous investigations also indicated that *M. loti* NZP2213 was capable of growing and surviving inside free-living amoebae of the *Acanthamoeba* genus, which resulted in its enhanced ability to develop nodules on *L. corniculatus* [8]. 

Lipopolysaccharides (LPSs) are a class of glycoconjugates unique to Gram-negative bacteria; they are present in the bacterial outer membrane (OM) and consist of three regions: lipid A, core oligosaccharide, and O-polysaccharide (O-PS, O antigen). High-molecular-weight LPS (S-LPS type), containing all of the above-mentioned components, is characteristic of smooth bacteria. The outer membrane of rough forms has no O antigen chains (R-LPS type) [9]. The two variants of bacteria, besides forming different colony phenotypes on solid media, vary in the activation of defense mechanisms in their eukaryotic hosts, plant and animal alike [10]. This is, among other things, due to the presence of a hydrophilic O-PS moiety in the S-LPS form, which comes into contact with the environment and provides a barrier against complement-mediated lysis, phagocytosis, and oxidative burst [11,12,13]. By limiting the permeability of the OM, LPS molecules protect the bacteria from different antibacterial agents, e.g., cationic peptides and some antibiotics [14]. In particular, O-PS appears to be the key moiety ensuring the survival and development of the bacteria residing inside the host.

Some studies show that protozoans can improve several functions of the soil microbiome relevant to plant health. First of all, they can directly influence the populations and the make-up of bacterial communities by grazing, thus indirectly enhancing bacterial functionality and ability to support plant growth in agricultural settings. Additionally, the presence of protozoa in the rhizosphere promotes plant growth by affecting the circulation of organic matter and the activation of bacterial genes needed, among others, for pathogen suppression. Living in close vicinity to Protists also exerts pressure on bacteria, which are forced to develop strategies to escape predation. A rapid coevolution may thus cause Protists to function as boosters for microbes introduced into the soil, ensuring their survival [15]. These interactions are clearly seen in *Acanthamoeba* spp., one of the most abundant functional groups of soil Protists, which on the one hand have been used as models for studying protozoan grazing [16], but, on the other hand, have been shown to act as hosts for many pathogenic bacteria, on which they exert selective pressure, resulting in the development of bacterial features which allow the pathogens to avoid clearance [17]. Knowing that the LPS molecule, and particularly its O-PS component, is one of the determinants involved in prey recognition and uptake [18], we wanted to explore the impact of the polysaccharide O chain on the cell surface properties of *M. loti* and their role in its survival within *A. castellanii*. To investigate these relationships, we used a previously chemically characterized NZP2213.1 mutant with a currently verified location of the transposon insertion within the *O*-acetyltransferase gene (*oatB*). As previously determined, the amount of O-PS in the mutant is reduced by half in comparison to the WT strain. It is composed of non-substituted as well as *O*-acetylated or *O*-methylated 6dTal*p* residues [7,19]. In turn, the O-chain of the WT strain isolated from the phenol-soluble LPS species was reported to be highly hydrophobic due to the occurrence of 2-*O*-acetyl-6dTal*p* exclusively [20]. For better understanding of the importance of O-PS in the bacteria-amoeba interactions and the cell wall features, a complementation mutant with a restored wild-type phenotype was constructed. The cell surface properties that possibly promoted the survival of *M. loti* inside amoeba cells were assessed using sensitivity assays, hydrophobicity and permeability tests of the bacterial envelope, and atomic force microscopy studies of the cell surface ultrastructure. The fate of the bacteria internalized by the amoebae was evaluated based on co-culture assays combined with fluorescence staining and electron microscopy studies. The combined data indicated that the smooth form of LPS was most probably responsible for the resistance of *M. loti* to amoeba grazing, unlike *O*-acetyl decorations, which were not involved in the induction of either oxidative or nitrosative stress, both of which play a role in bacterial clearance.

## 2. Results

### 2.1. Clarification of the Localization of the Tn5 Insertion in the NZP2213.1 (oatB) Mutant in the Light of New Data

In search of the function of the gene disrupted in the previously characterized NZP2213.1 mutant, we used a sequence near the transposon integration site, established in an earlier study, as a query to look for similar sequences within the available database of genomic sequences. The most similar sequence was found in the genome of *Mesorhizobium ciceri* bv. *biserrulae* WSM1284 within the A4R29_08460 gene, which encoded a hypothetical *O*-acetyltransferase. The genomic sequence of this strain was used to design primers for amplification of the gene from the wild-type *Mesorhizobium loti* NZP2213 genome. The amplification was successful, and the obtained amplicon was used to construct a complementation plasmid. Sequencing of the resultant plasmid showed that the gene, named *oatB* (GeneBank No.MH626640), shared 93% identity with the A4R29_08460 gene from *Mesorhizobium ciceri* bv. *biserrulae* WSM1284. BlastP searches revealed that the most similar hypothetical proteins with the predicted function of *O*-acetyltransferases were encoded within the *Mesorhizobium loti*, *Mesorhizobium ciceri* (99%/100% and 93%/96% identity/similarity, respectively), and *Mesorhizobium* sp. L103C119B0 (88%/94% identity/similarity) genomes.

**Figure 1:**
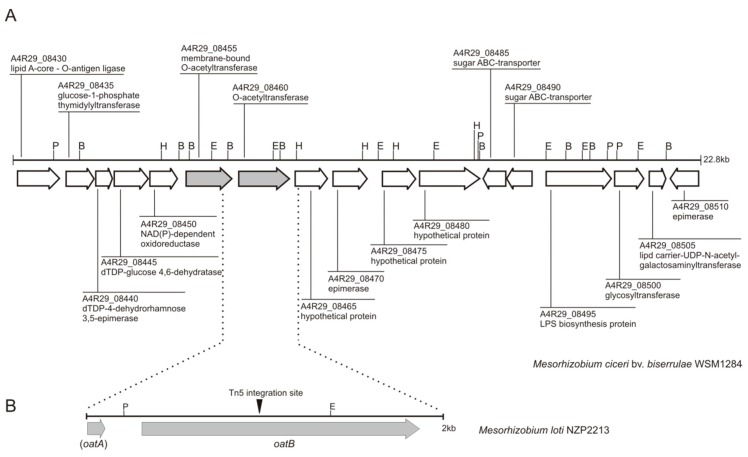
Physical and genetic map of: (**A**) a chromosomal region of *M. ciceri* bv. *biserrulae* WSM1284 grouping genes implicated in lipopolysaccharide biosynthesis and polymerization. The sequences flanking the A4R29_08460 gene were used to design primers for amplification of the corresponding fragment from the chromosome of *M. loti* NZP2213. Hypothetical functions of gene products come from a GenBank annotation, except for the A4R29_08455 and A4R29_08460 genes, the function of which was predicted in this work on the basis of similarity of their amino acid sequences to proteins found in genome databases; (**B**) a chromosomal region of *M. loti* NZP2213 covering the 3′-end of the *oatA* gene and the whole *oatB* gene, which was cloned into the broad-host range vector and used to complement the NZP2213.1 transposon mutant described previously [7]. Marked is the Tn5 integration site within the *oatB* gene. P: PstI, B: BamHI, H: HindIII, and E: EcoRI.

In the *M. ciceri* bv. *biserrulae* WSM1284 genome, the *O*-acetyltransferase-encoding gene is located in the region where other genes implicated in LPS synthesis and modification have been annotated. Bioinformatic analyses of the amino acid sequence of the hypothetical OatB *O*-acetyltransferase revealed the presence of putative transmembrane helices (Figure 1). 

The plasmid carrying the DNA insert with the *oatB* gene, also containing the 3′-end of the preceding *oatA* gene encoding yet another hypothetical *O*-acetyltransferase, was introduced into NZP2213.1 mutant cells. The resulting NZP2213.1(pBGoatB) strain was tested for LPS phenotype and *O*-acetylation of the 6dTal*p* residues building the O-PS.

### 2.2. Restoration of O-acetyl Transferase Function in the Complemented Strain. LPS and O-PS Phenotype

^1^H nuclear magnetic resonance (^1^H NMR) analyses of O-PSs from WT, mutant NZP2213.1, and complemented NZP2213.1(pBGaotB) strains were performed to provide information about the function of the oatB gen as *O*-acetyl transferase.

**Figure 2:**
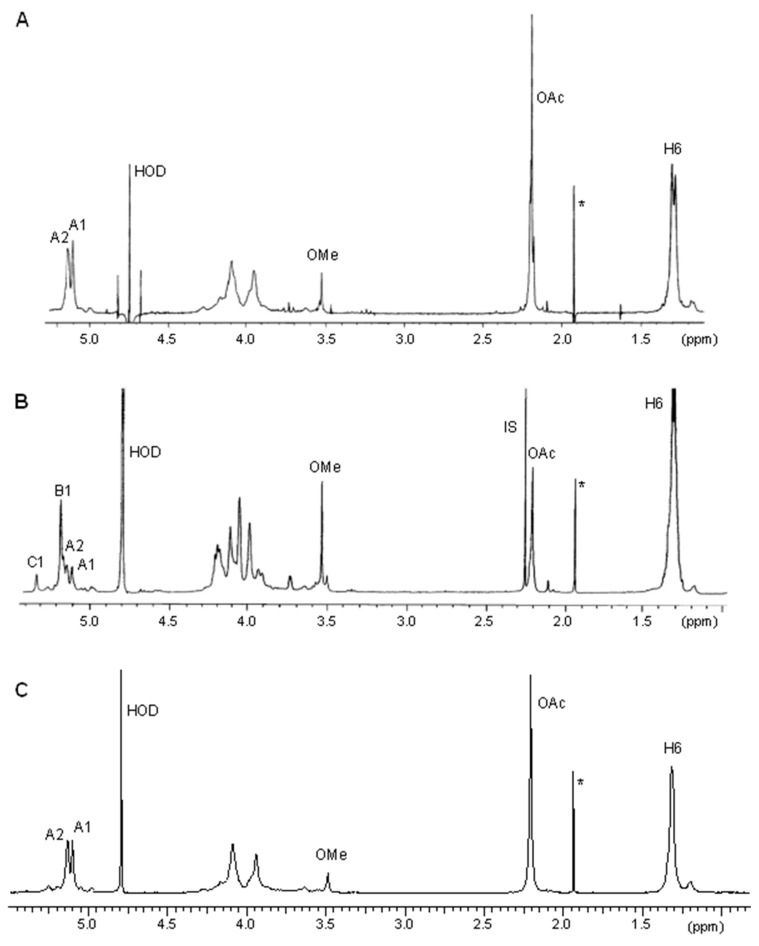
^1^H NMR spectra of the O-PSs of *M. loti* WT (**A**), NZP2213.1 mutant (**B**) and complemented (**C**) NZP2213.1(pBGoatB) strains. The O-PS samples were examined in 99.98% D_2_O using acetone (δ_H_ 2.225) as an internal (**B**) or external standard (**A**,**C**). **A**1—H-1 of 2-*O*-acetyl-6-deoxytalose (δ_H_ 5.1); A2—H-2 of 2-*O*-acetyl-6-deoxytalose (δ_H_ 5.12–5.14); B1—H-1 of 6-deoxytalose (δ_H_ 5.17); C1—H-1 of 2-*O*-methyl-6-deoxytalose (δ_H_ 5.31); OAc—*O*-acetyl methylprotons (δ_H_ 2.19–2.2); OMe—methyl protons (δ_H_ 3.49–3.54); H6—signals of methyl protons of 6-deoxytalose residues in the range of δ_H_ 1.28–1.31; IS—indicates internal acetone; *—proton signals of free acetic acid (δ_H_ 1.93).

The O-PSs of the studied strains were obtained by mild acid hydrolysis of the phenol-soluble S-LPS species followed by removal of a lipid A portion by centrifugation and fractionation of the supernatants by GPC (gel permeation chromatography) on Sephadex G50 fine. High-molecular-weight fractions representing the O-chain were then compared in ^1^H NMR experiments (Figure 2). Two major signals were observed in the low-field region of the ^1^H NMR spectrum of the NZP2213.1(pBGoatB) O-PS (Figure 2C). The first one, at δ 5.1, was the anomeric proton resonance of 6dTal*p*; and the second signal, at δ 5.14, belonged to a H-2 signal, shifted downfield due to 2-*O*-acetylation of the 6dTal*p* residue (β-effect of *O*-acetylation), as similarly observed in the ^1^H NMR spectrum of the wild-type O-PS (Figure 2A). The calculation of the integration values for the proton signals of *O*-acetyl groups at δ 2.2 to that of the anomeric proton indicated stoichiometric *O*-acetylation of the 6dTal*p* residues in the O-PS of the complemented NZP2213.1(pBGoatB) strain. On the other hand, in the anomeric region of the ^1^H NMR spectrum recorded for O-PS of the NZP2213.1 mutant, three out of four observed signals at δ 5.1, 5.17 (the major one), and 5.31 were assigned to H-1 protons of 2-OAc-6dTal*p*, 6dTal*p*, and 2-*O*-methylated 6dTal*p*, respectively, and the last one at δ 5.12 to H-2 of 2-OAc-6dTal*p* (Figure 2B).

In the previous report, it was shown that the NZP2213.1 mutant is resistant to lysis of phage A1 due to the changed LPS structure, and fully *O*-acetylated wild-type O-PS was indicated to be a receptor for the phage [19]. In the present study, the complemented NZP2213.1(pBGoatB) strain subjected to the action of phage A1 was lysed similarly to the WT strain.

Thus, the combined data from the ^1^H NMR experiments and phage A1 sensitivity confirmed the restoration *O*-acetyltransferase function in the O-PS of the complemented NZP2213.1(pBGoatB) strain.

The complementation of *O*-acetyltransferase function in the NZP2213.1(pBGoatB) strain not only resulted in substitution of 6dTal*p* with *O*-acetyl groups, but also led to an increase in the content of HMW, S-LPS fraction in the OM, to the wild-type level. This might suggest the pleiotropic effect of the mutation in the oatB gene, which was established on the basis of the yield of phenol-soluble S-LPS species, SDS-PAGE, and the sugar composition analysis of O-PSs.

The yield of phenol-soluble S-LPS species extracted from the NZP2213.1 mutant cells was lower than for WT. In turn, the yield of lipophilic S-LPS obtained from the cells of the complemented NZP2213.1(pBGoatB) strain was restored up to the level of the WT strain. Additionally, the amount of S-LPS in OM of the NZP2213.1 mutant was diminished by half [7], and the content of S-LPS in the complemented strain was restored, as confirmed by the SDS-PAGE (Figure S1).

The molar ratio of the main sugars building the O-chain i.e., 6-deoxytalose (6dTal*p*) and 2-*O*-methyl-6-deoxytalose (2-OMe-6dTal*p*), in relation to *N*-acetylquinovosamine (QuipNAc), which is a sugar of the outer core tetrasacharide [19,21], was ~33:1 and 15:2, in lipophilic S-LPS of the WT and mutant NZP2213.1 strains, respectively. In turn, the molar ratio of 6dTal*p* and 2-OMe-6dTal*p* estimated at ~27:1.9 in the O-PS of the complemented NZP2213.1(pBGaotB) strain indicated the restoration of the WT proportion of sugars (Table 1).

### 2.3. Physicochemical and Surface Properties of the NZP2213.1 (oatB) Mutant

Since cell surface hydrophobicity plays a crucial role in the attachment of bacteria to host cells, and this factor depends, among other things, on the structure and abundance of LPS, we determined adhesion values for the studied strains using a two-phase system. The data showed that adhesion to dodecane was similar for the WT, NZP2213.1(pBGoatB), and NZP2213.1 mutants at 28.07 ± 0.9, 25.13 ± 1.5, and 22.35 ± 5.1, respectively, and the differences were not statistically significant. 

**Figure 3:**
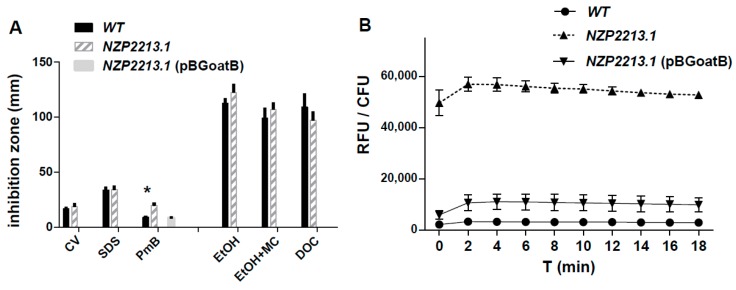
Surface properties of the *M. loti* cell wall. (**A**) Sensitivity assays; (**B**) *N*-phenyl-1-naphtylamine uptake assay. Abbreviations: CV—crystal violet, SDS—sodium dodecyl sulfate, PmB—polymyxin B, EtOH—ethanol, MC—divalent cations (Mg^2+^ and Ca^2+^). Data were analyzed with Graph Pad software using Students’s *t-*test. * *p* < 0.05.

A sensitivity assay with the use of some detergents, known cell envelope-disrupting agents, and the peptide antibiotic polymyxin B (PmB), demonstrated that, generally, the NZP2213.1 mutant was not more sensitive to the membrane-disrupting agents than WT (Figure 3A). The only exception was its higher sensitivity to polymyxin B (*p* < 0.05). The integrity of the OM was also tested with a hydrophobic 1-*N*-phenylnaphthylamine (NPN) probe, which cannot enter an intact OM, but can pass through a destabilized one, giving rise to strong fluorescence. In the NPN uptake assay, we established that the WT strain exhibited a 17-fold lower level of fluorescence, calculated as relative values (RFU/CFU), in comparison to the NZP2213.1 mutant (*p* < 0.0001) (Figure 3B).

**Figure 4:**
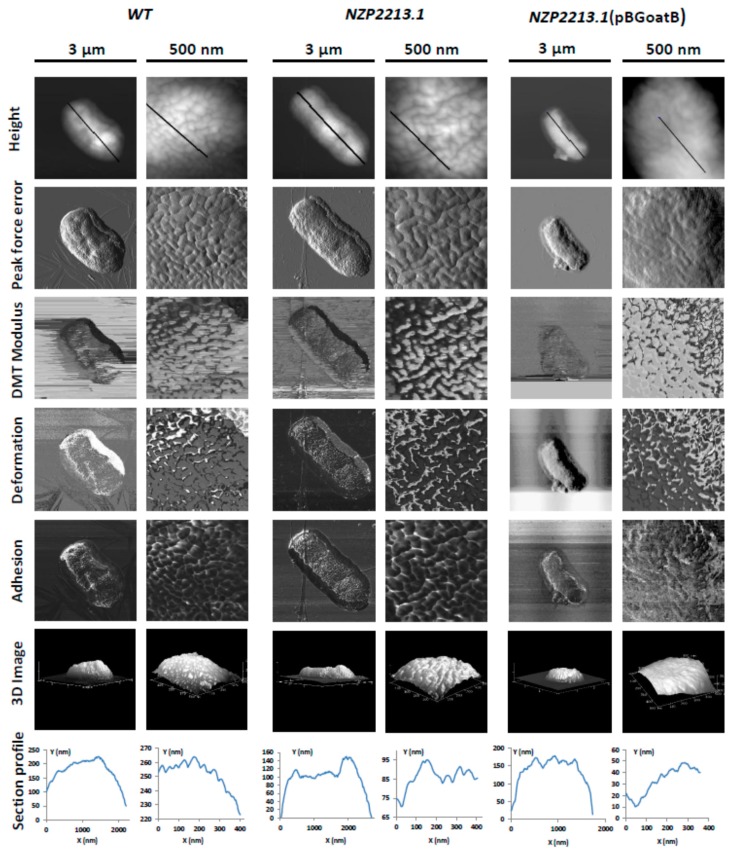
Atomic force microscopy (AFM) images of wild-type *Mesorhizobium loti* NZP2213 (WT), the NZP2213.1 mutant, and the complemented NZP2213.1(pBGoatB) strain. The columns show air-dried whole cells (3 × 3 µm) and surface ultrastructures at a high resolution (500 × 500 nm). Section profiles, presented in the lower panel, correspond to the height profiles (upper panel) obtained along the black scan lines shown in them.

The structural basis for the permeability of OM in *M. loti* strains was studied using atomic force microscopy. The topography changes and the surface ultrastructure of wild-type *M. loti*, the NZP2213.1 mutant, and its complemented strain NZP2213.1(pBGoatB) were investigated by observing cells deposited on bare mica discs, dried in ambient conditions and scanned in air by AFM (Figure 4). All tested strains had the same rod-shaped morphology typical of *M. loti*, but they varied in size. The average length and width of WT and NZP2213.1(pBGoatB) cells was 2.21 ± 0.045/1.05 ± 0.004, and 2.03 ± 0.25/0.878 ± 0.05 µm, respectively. The cells of the NZP2213.1 mutant were longer (*p* < 0.05) and measured 2.74 ± 0.151/1.093 ± 0.038 µm. The evaluation of surface roughness of bacterial cells, which is a quantitative assessment of surface texture, defined as the average of the vertical deviations from the nominal surface over a specified surface area, showed that the average root-mean-square roughness (RMS) of the NZP2213.1 cells was slightly higher than that of the WT strain, and similar to NZP2213.1(pBGoatB) (3.03 ± 0.65 nm, 2.72 ± 0.46 nm (*p* < 0.05), and 3.12 ± 0.56, respectively). Since RMS makes no distinction between peaks and valleys, and we obtained equal RMS values for the mutant and its complemented strain, we carried out a thorough analysis of waviness, which is the measure of the more widely spaced component of surface texture [22]. The section profiles of bacteria obtained along their long cell axis showed that the waviness heights for WT and NZP2213.1(pBGoatB) were much larger than for the NZP2213.1 mutant. Additionally, WT and the complemented strain had lenticular profiles, while all the observed NZP2213.1 mutant cells were flattened in the middle (Figure 4). 

The surfaces of the WT and NZP2213.1(pBGoatB) strains were regularly granular and had densely packed oval subunits composed of aggregates of surface molecules. By contrast, the surface of the mutant was more varied and had less well-defined, but statistically larger (*p* < 0.001), elongated and curved units with loose lateral packing. The analysis of the section profiles of the NZP2213.1 mutant revealed that its irregular units were separated by 4.5–21.0 nm-deep depressions. Similar dents between the units of the WT and NZP2213.1(pBGoatB) strains were shallower, and their depth ranged between 1.8–9.2 and 0.4–8.6 nm, respectively. 

Apart from the differences in the topography of the NZP2213.1 mutant, its nanomechanical properties were also altered when compared to WT and NZP2213.1(pBGoatB), as shown in the peak force error, DMT (Derjaguin, Muller and Toporov) modulus, adhesion, and deformation images (Figure 4). The measurement data showed that the surface of the NZP2213.1 cells was more inflexible, as reflected by a 1.3-fold increase in the DMT modulus relative to the values obtained for the WT strain (NZP2213.1 = 7.726 ± 6.26, WT = 5.867 ± 0.089, NZP2213.1(pBGoatB) = 6.517 ± 1.44 GPa). The DMT modulus values for the mutant exhibited high standard deviations, probably because of an uneven distribution of O-PS residues in the middle part of the cells in which the measurements were taken [23].

### 2.4. The Influence of Mutation in the oatB Gene on the Interactions between M. loti and A. castellanii

**Figure 5:**
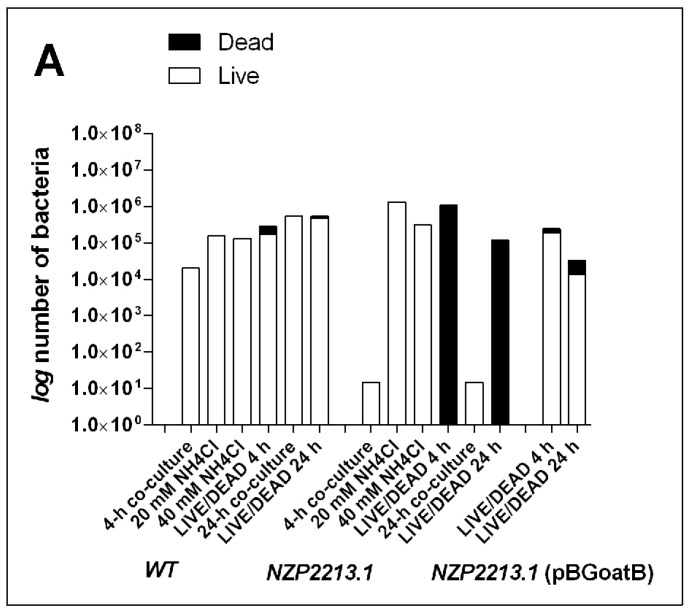

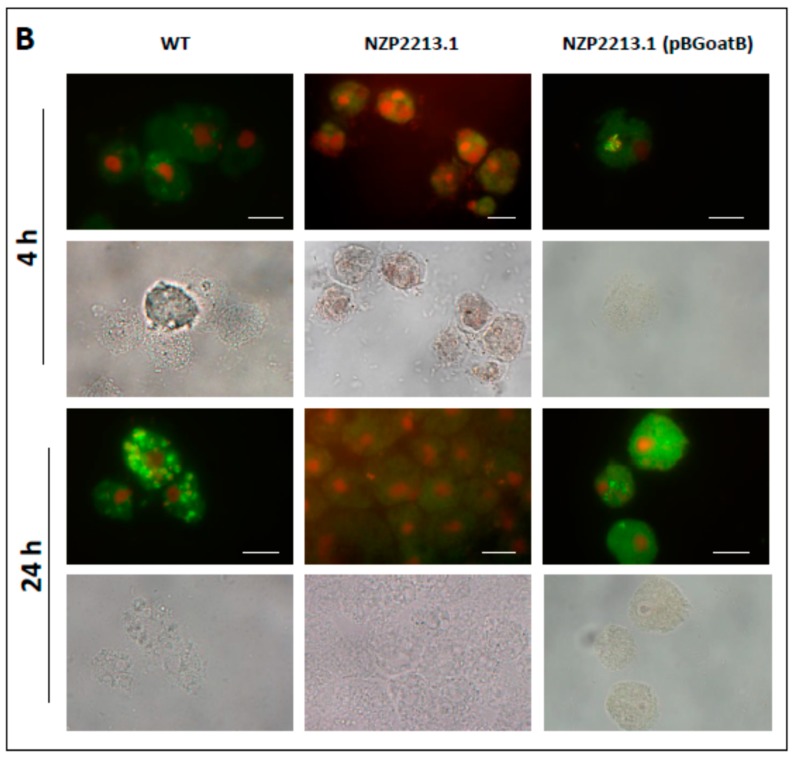
*M. loti*–*A. castellanii* co-culture assays. (**A**) Graphs represent average counts of *M. loti* cells internalized by amoebae per one well of a tissue culture plate. CFU counts of viable bacteria were estimated for 4- and 24-h co-culture assays and for phagolysosome inhibition assays, in which NH_4_Cl was used to alkalinize phagolysosome compartments. In the LIVE/DEAD assays, the total number of internalized bacteria (viable and non-viable) was calculated from registered images representing at least 200 randomly selected amoebae cells. (**B**) Differential LIVE/DEAD fluorescence staining of *M. loti* within *A. castellanii* vesicles after 4-h and 24-h co-incubation. Green fluorescence indicates metabolically active bacterial cells with an intact membrane; the red-stained bacteria are considered metabolically inactive. Images were collected on a Zeiss LSM780 laser scanning confocal microscope with excitation light using dual color channels (upper panels). The same samples were also registered using transmitted light (lower panels). Bars represent 10 µm. Details of the experiments are described in Section 4.

To investigate whether the reduced amount of O-PS in the outer membrane of *M. loti* affected bacterial internalization and survival within *A. castellanii*, the NZP2213.1 mutant, the WT strain, and the complemented NZP2213.1(pBGoatB) strain were used in co-culture assays. The counts of live intracellular bacteria were estimated using invasion assays after 4-h and 24-h incubation of the bacteria with the amoebae. The titers of internalized bacteria for the NZP2213.1 mutant were much lower than for the WT strain (Figure 5A), for both the short-term and the long-term interactions. Since it was not clear whether the small remnants of mutant cells recovered alive from the amoebae at the tested time points were a result of poor internalization or intense digestion of the bacteria by the amoebae, the preparations from the invasion assays were stained with the LIVE/DEAD kit (BacLight kit, Invitrogen by Thermo Fisher Scientific, Waltham, MA, USA) or tested using the Gimenez method to visualize the overall number of internalized bacteria. The images (Figure 5B) were used to calculate the total number of intracellular bacteria and the number of bacteria per single amoeba cell, and to evaluate what part of the *A. castellanii* population was infected by the bacteria. Estimates showed that after 4-h co-incubation, 79.26 ± 8.0, 35.06 ± 5.8, and 52.23 ± 1.58% of *A. castellanii* cells were infected with the NZP2213.1 mutant, WT, and NZP2213.1(pBGoatB) strains, respectively. After the 24-h assay, the corresponding numbers were 42.14 ± 2.4, 66.24 ± 1.3, and 19.86 ± 0.1%. The average number of internalized bacteria per amoeba after 4 h of incubation was much higher for the NZP2213.1 mutant than for WT and NZP2213.1(pBGoatB) (8.89 ± 2.5, 1.84 ± 1.5, and 1.56 ± 3.2, respectively), but after 24 h, it dropped, while the number of internalized WT bacteria increased (2.02 ± 1.1, 2.74 ± 1.5, and 0.17 ± 0.5 respectively). These data showed that despite the fact that the rate of internalization of the NZP2213.1 mutant was the highest at the beginning of the co-culture assays, its population was quickly digested by the endocytolytic pathway (Figure 5). After 24 h of co-culture, only single metabolically inactive mutant bacterial cells were observed inside *A. castellanii* cells. By contrast, metabolically active WT and NZP2213.1(pBGoatB) cells were observed inside the amoebae after both the 4-h and the 24-h co-culture assay (Figure 5B).

Since the LIVE/DEAD test is known to have many limitations, and, additionally, in our experiment, the fluorescent stains from the kit had to penetrate through two membrane systems (of the host and the bacteria), we performed a phagocytosis inhibition assay to better assess the intra-amoebic killing of *M. loti* strains. In the assay, NH_4_Cl was used for the alkalinization of acidic compartments leading to the inhibition of the endocytolytic pathway, to avoid net movement of charge across the endosomal-limiting membrane involved in bafilomycin-induced alkalinization [24]. In the experiment, the counts of live bacteria recovered from amoebae pre-treated with NH_4_Cl, in relation to non-treated amoebae, increased by 5 and 1 log for the NZP2213.1 mutant and the WT strain, respectively. This clearly showed that prevention of phagolysosome fusion significantly increased the possibility of survival of the mutant, thus confirming the data from the LIVE/DEAD and co-culture assays (Figure 5).

**Figure 6:**
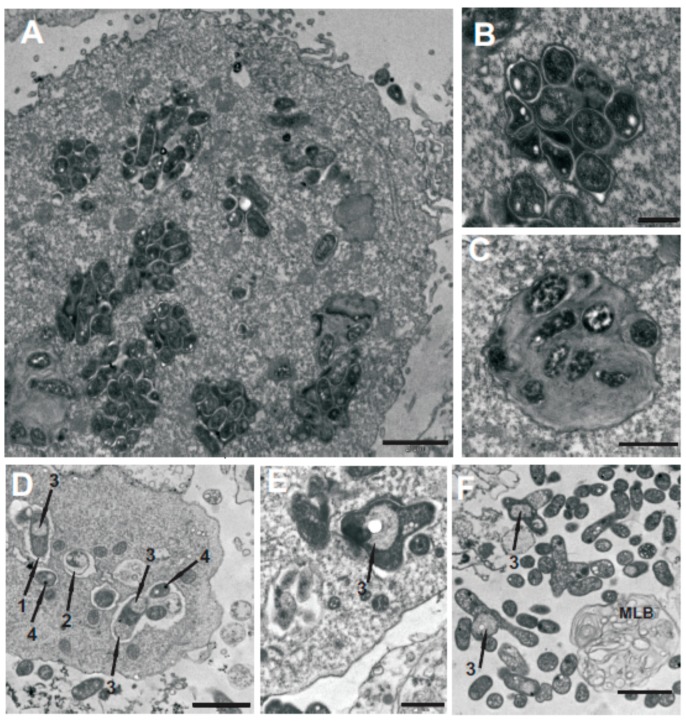
24-h co-culture assays. Transmission electron microscopy (TEM) images of *A. castellanii* filled with *M. loti* cells: (**A**–**C**) WT, (**D**–**F**) NZP2213.1 mutant. Scale bars: 2 µm (**A**,**D**,**F**), 1 µm (**E**), and 500 nm (**B**,**C**). Arrows: 1—membrane ruffling, 2—membrane detachment, 3—cytoplasm release, 4—electron-dense dots. Abbreviation: MLB—multilamellar body.

The ultrastructural localization and morphology of the bacteria inside *A. castellanii* were monitored by transmission electron microscopy (TEM) after 24-h co-culture. The micrographs revealed that, for both NZP2213.1 and WT, single bacterial cells rather than clusters of bacteria had been taken up by endocytosis into vacuoles [25]. It appeared that the morphology of the internalized NZP2213.1 cells was changed, with observable membrane ruffling and/or detachment, formation of electron-dense dots inside the cells, and release of cytoplasmic material [26]. Electron micrographs also showed that the mutant cells were enclosed inside spacious *Mesorhizobium*-containing vacuoles (MCVs), and some of them were branched. Outside the amoebae, there were multilamellar bodies (MLBs) which were empty or contained single NZP2213.1 cells. In the case of the WT strain, most MCVs were tightly packed with morphologically unaltered cells (Figure 6).

Bacteria become associated with amoebae by interacting with various lectins [27,28,29,30]. In this and earlier studies, we showed that mannose strongly inhibited (99.8 ± 5.5%) (*p* < 0.0001) the association of WT with amoeba cells. For the NZP2213.1 mutant, the mannose-inhibited association was 5-fold lower (19.0 ± 4.4%). Gal/Rha-mediated blocking was in the range of 28.0–32.0% for both strains. 

### 2.5. Bactericidal Mechanism of Clearance of the NZP2213.1 (oatB) Mutant by A. castellanii 

The fast and efficient internalization of NZP2213.1 cells by *A. castellanii* ultimately led to their clearance from the amoebae. This observation raised the question of what bactericidal mechanism was involved in this effective elimination of the mutant cells. As a response to bacterial invasion, phagocytic cells can generate reactive nitrogen species (RNS) and reactive oxygen species (ROS). Nitric oxide (NO) is produced by inducible nitric oxide synthase (iNOS), and superoxide anion radicals (O_2_^−^) by NADPH oxidase (NOX) [31,32]. 

**Figure 7:**
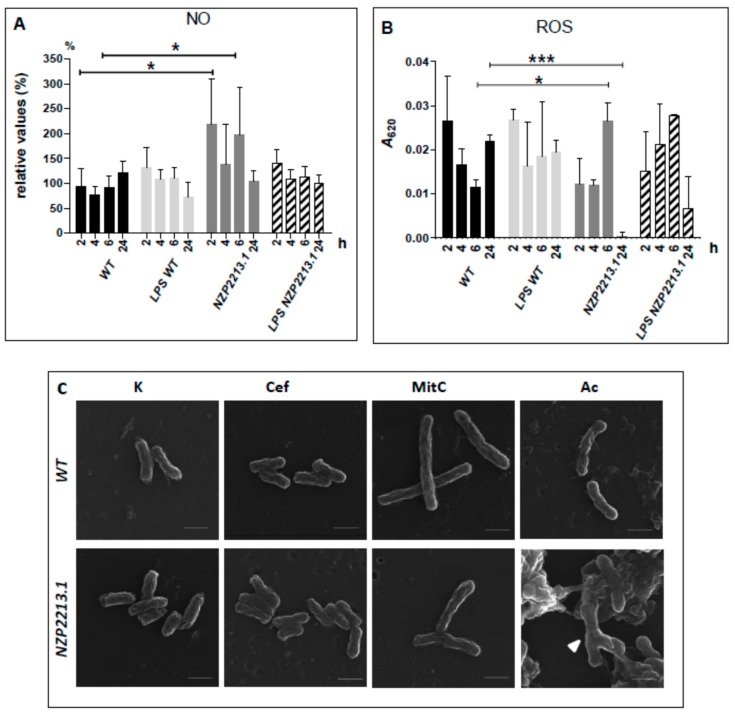
Summary of bactericidal mechanisms used by *A. castellanii* against *M. loti*. Graphs show average production of (**A**) NO_2_^−^, and (**B**) phagocytic ROS (O_2_^−^) by amoeba cells activated with whole WT or NZP2213.1 mutant cells or LPSs derived from these bacterial strains; the amounts are shown per one well of a tissue culture plate. The production levels of NO_2_^−^ in the culture supernatant were determined by the Griess reaction using a calibration curve for NaNO_2_. Liberation of ROS was estimated on the basis of nitro blue tetrazolium (NBT) reduction to formazan particles (details described in Section 4). Data were analyzed with Student’s *t-*test using Graph Pad software. * *p* < 0.05, *** *p* < 0.001. (**C**) SEM (scanning electron microscopy) images of the effect of cephalexin (Cef), mitomycin C (MitC), and amoeba lysate (Ac), in acidic conditions (50 mM acetate buffer pH 5.0), on the morphology of WT and NZP2213.1 cells, compared to untreated cells (K). Scale bars: 1 µm. The white arrow indicates the position of the Y-shaped cell.

To assess NO generation by *A. castellanii*, the concentrations of nitrite (a stable nitric oxide breakdown product) were measured using the Griess assay. Amoeba cells were stimulated using whole NZP2213.1 or WT bacteria or LPSs derived from these two strains. The results were compared to those obtained for non-stimulated control amoeba cells (100%). The data are presented in Figure 7A as a percentage of the control values. An analysis of the data showed that NO generation by amoebae was strongly induced only in the presence of NZP2213.1 mutant cells; nearly two-fold elevated levels of this compound were determined after 2 and 6 h of co-incubation (*p* < 0.05).

ROS production was estimated on the basis of the levels of O_2_^−^ measured spectrophotometrically by detection of formazan created after the reaction of nitro blue tetrazolium (NBT) with O_2_^−^. The liberation of ROS during phagocytosis was calculated from data obtained for bacteria- or LPS-challenged amoebae pre-treated or not (total intracellular ROS) with iodoacetamide (an inhibitor of phagocytosis). The concentrations of phagocytic ROS produced upon stimulation of amoebae with NZP2213.1 cells were statistically higher than in the case of WT cells at 6 h p.i. After 24 h of incubation, however, ROS values registered for NZP2213.1-challenged amoebae were much lower in comparison to those obtained for WT-challenged amoebae (*p* < 0.001). This was presumably connected with an almost complete clearance of the mutant, as established earlier by the LIVE/DEAD assay. Since the levels of ROS produced upon stimulation with whole cells were similar to those obtained upon stimulation with LPS alone, it seems very plausible that ROS was mostly generated in response to LPS moieties (Figure 7B).

To establish the impact of hydrogen peroxide (another form of ROS) on the survival of NZP2213.1 and WT cells, we subjected them to treatment with 10 mM H_2_O_2_ for 10 min, followed by CFU determination. The results showed that both strains were equally influenced by H_2_O_2_, which reduced their titers by 48.1 ± 10.7 and 46.6 ± 13.0%, respectively.

It is a well-established fact that amoebic lytic enzymes can clear Gram-positive, but not Gram-negative, bacteria because in the latter, the LPS layer, being an efficient barrier of permeability simultaneously protects target sites against the action of enzymes [33]. Since the NZP2213.1 mutant, characterized by incomplete substitution of lipid A-core moieties with O-PS, was more permeable to both polymyxin B and fluorescent NPN than the WT strain, we concluded it was also more permeable and, hence, more sensitive to *A. castellanii* lytic enzymes. Another observation that supported this conclusion were the vast differences in the morphology of the NZP2213.1 and WT cells internalized by amoebae, as visualized by TEM. To confirm our supposition, we subjected both strains to the action of amoeba lysate devoid of nuclei and mitochondria, in acidic conditions typical of lytic compartments. To exclude the effect of non-lysosomal factors, a control study was carried out under normal conditions. The morphology of treated bacteria was examined using SEM. NZP2213.1 cells, but not WT cells, and only those treated in the acidic environment, displayed buds, swellings and branches (Figure 7C), which resembled those observed in TEM-micrographs. Such changes are often associated with a cell cycle arrest or inhibition of cell wall synthesis. To dispel the doubts, we treated both strains with the cytostatic mitomycin C and with cephalexin, a cell division-blocking agent [34]. While cephalexin did not have any visible impact on the shape of the bacteria, 24-h treatment with mitomycin C generated branching of some NZP2213.1 cells and elongation of WT cells (Figure 7C). 

It is known that acidification of endosomes in amoebae occurs soon after initial endocytosis and a drop below pH 5.0, after which the pH may return to nearly neutral [35]. The ability of *M. loti* strains to grow in the pH range of 4.5–7.0 was tested in broth cultures as a change in the optical density during 24-h culture, confirmed by plating on 79CA medium. The results of the study showed no differences in the growth of the NZP2213.1 mutant and the WT strains in the tested pH range. 

## 3. Discussion

Lipopolysaccharide is a glycoconjugate found in the envelope of Gram-negative bacteria. It mainly plays a protective function, but also participates in the triggering or inhibition of defense mechanisms in eukaryotic cells. The lipid A portion of LPS, which is capable of ligating to Toll-like receptors (TLRs) in higher organisms, is mostly responsible for immunomodulatory properties of the glycoconjugate, while the O-PS moiety can participate in the subversion of cell-autonomous host defense mechanisms such as phagocytosis, autophagy, oxidative and nitrosative stresses, and the action of extracellular traps and antimicrobial peptides [36,37]. Various observations suggest that a proper amount of high-molecular-weight LPS species in the outer membrane is required for invasion and survival in both symbiotic and parasitic microbe–host interactions in plant, animal, and amoeba models [37,38,39,40]. The chemical composition and structure of the O-antigen are of great importance as well [10,41].

In our previous report, we showed that between two well-characterized *M. loti* strains capable of entering into symbiosis with the *Lotus* group species, MAFF303099 and NZP2213, only the latter was able to resist grazing by the ubiquitous amoeba *A. castellanii*. The interaction with the Protist enhanced the ability of the bacterium to develop more nodules on *L. corniculatus*, which suggested it could possibly be used as a plant inoculant [8]. However, the molecular mechanisms involved in this interaction were not determined. A proper amount of smooth LPS in the outer membrane of this strain had been shown earlier to be crucial for symbiosis with the host plant [7]. In the present study, to establish the determinants of *M. loti*–*A. castellanii* interactions, we used the same NZP2213.1 mutant, whose O-PS was reduced by half and was not entirely substituted with *O*-acetyl groups [19]. The region of this mutant interrupted by a transposon had been characterized previously and shown to display a low similarity to a membrane glycosyltransferase gene of *Caulobacter crescentus* (CB15, 43%/53% identity/similarity) [7]. The currently verified location of the insertion (based on the new data) mapped the mutation to the *oatB* gene (with the predicted function of *O*-acetyltransferase) in the gene cluster involved in the synthesis and assembly of the O-antigen. This region showed 93% identity with the A4R29_08460 gene from *M. ciceri* bv. *biserrulae* WSM1284. The function of the *oatB* gene as *O*-acetyltransferase was confirmed by ^1^H NMR spectroscopy, which revealed a lower degree of substitution of 6dTal*p* residues with *O*-acetyl groups for the NZP2213.1 mutant and restoration of their abundance in complemented NZP2213.1(pBGoatB) as for the WT strain. As a result, the complemented strain exhibited similar A1 phage sensitivity as the wild-type, in contrast to the NZP2213.1 mutant. Additionally, in the previous report, on the basis of the yields of LPSs from water and phenol phases, SDS-PAGE, and sugar composition analysis (the molar ratio of the sugars representing the O-antigen in relation to the outer core sugar), it was indicated that the NZP2213.1 mutant has a diminished amount of the O-antigenic determinant in LPS [7,19]. Similar analysis for the complemented strain indicated that the amount of S-LPS was restored to the level observed for the WT strain. These arrangements were additionally confirmed by images of cell surfaces registered with the use of the AFM microscopy, a technique that was successfully used by others to characterize the phenotype of mutations within genes responsible for the cell envelope synthesis and function [42,43]. The examination of 3D topographic AFM images of the *oatB* mutant revealed changes in the size of the surface units, representing tightly packed LPS molecules and depressions separating them. The patches containing LPS moieties were more irregular and bigger and the spaces between them were deeper and wider than those observed in the WT and complemented strains. The changes caused by the mutation within the *oatB* gene were somewhat similar to those induced by metal depletion from OM of *Escherichia coli* with EDTA treatment and resulting in removal of some LPS molecules [44].

Thus, the phenotype of the mutant indicated that the *oatB* gene might play a dual role: in the *O*-acetylation of L-6dTal*p* residues and in the assembly of the O-antigen. Thomsen et al. revealed that the transposon mutagenesis of the *manC* gene of *Salmonella enterica*, encoding glycosyltransferase, affected not only its expression but also two downstream genes, resulting in the reduction of the amount of S-LPS produced by the mutant, compared with the wild-type strain [37]. Likewise, an insertion of an IS*1*-like element into the putative acetyltransferase WclK gene of *Escherichia coli* influenced both acetylation and O-PS synthesis [45]. 

Mutations within the O-antigen cluster usually eliminate an entire O-antigen chain, creating mutants that exhibit pleiotropic phenotypes: changed physicochemical cell surface properties, loss of mobility, or reduced tolerance to osmotic pressure, high temperature, acidic pH or oxidative stress. These deficiencies result in impaired survival within host cells [13,37,40,46]. Since O-PS plays a crucial role in resistance to toxic agents and antibacterials, it is obvious that deterioration in the synthesis of this moiety, leading to a rough phenotype, has far-reaching effects [13,41]. However, it has been shown that much smaller changes in LPS structure, such as shortening of the O-antigen chain and a reduced amount of intact O-PS can result in a higher susceptibility of Gram-negative bacteria to complement killing and negatively affect both their stress tolerance and virulence, respectively [37,47]. In this study, the inactivation of the *oatB* gene of *M. loti* increased the sensitivity of the mutant strain to hydrophobic compounds such as polymyxin B (Figure 3A) and enhanced the permeability of the OM to the fluorescent dye *N*-phenyl-1-naphthylamine (Figure 3B). PMB is known to interact mostly with the lipid A moiety, which means that the hydrophilic O-PS protects the cell against the penetration of this antibiotic into the deeper layers of the outer membrane [48]. Our AFM investigations showed that the O-antigen moieties of the *oatB* mutant were characterized by a lower density (Figure 4), and thus provided a weaker barrier against detrimental hydrophobic factors. Furthermore, as observed by contrast phase microscopy and SEM microscopy (Figure 7C), cells with depleted amounts of OatB, treated with amoeba lysate (containing lytic enzymes), exhibited gross morphological defects, including severe branching. A similar branched phenotype was observed in *Rhizobium meliloti* cells, and, in the present study, in the *oatB* mutant, after cell division had been blocked with the cytostatic antibiotic mitomycin C [34]. According to previous research, *A. castellanii* lytic enzymes are active against Gram-positive bacteria in the acidic environment of phagolysosomes, but are not able to penetrate the OM of Gram-negative microorganisms to reach their targets [33]. They can only do so when the continuity of the OM is disrupted [44]. The TEM study of trophozoites showed that, unlike the wild-type cells, the *oatB* cells internalized by amoebae and enclosed inside their vacuolar structures were misshapen, had aberrant bulges and a detached and ruffled membrane, and showed clumping of cytoplasmic material (Figure 6D–F).

In unicellular organisms such as amoebae, phagocytosis has developed as a trophic mechanism; however, some bacteria have created survival strategies that interfere with phagocytic internalization and/or maturation processes, thus helping these microorganisms to resist predation. The resistance mechanisms involve modulation of the rate of internalization, prevention of ligation with receptors, or modification of the phagosome membrane into a non-fusogenic parasitophorous vacuole providing a protective niche.

Bacterial adhesion to host cells is the first step for colonization to succeed. Initially, it is driven by a nonspecific physical interplay between two surfaces, among others by hydrophobic interactions, which result in reversible attachments. One of the surface structures of Gram-negative bacteria, which apparently can influence hydrophobicity, is lipopolysaccharide. This parameter is dependent on the O-PS or the lipid A parts of the LPS molecule, but not the core [49,50]. The structural differences within the O-PS chain that affect hydrophobicity include the glycosyl composition and sugar chain length as well the presence and saturation of non-carbohydrate substituents (methyl and/or acetyl groups) [10]. The longer O-PS and the more methyl and/or acetyl groups attached to it, the higher the hydrophobicity of the bacterial surface. The data obtained in our investigations showed that this parameter determined for the WT, NZP2213.1 mutant, and complemented NZP2213.1(pBGoatB) strains was statistically insignificant. This may seem a little surprising, considering the much lower content of *O*-acetyl groups in O-PS of the *oatB* mutant in comparison to the WT and complemented strains (Figure 2). However, their absence is partly compensated by the expression of *O*-methyl groups substituting 6dTal*p* residues [18]. Probably due to the presence of *O*-methyl groups, the hydrophobicity of the mutant was almost equal to that of the WT and complemented strains with fully *O*-acetylated O-PSs. It is well documented that some rhizobial LPSs undergo modification observed as addition of *O*-methyl group/s in the O-PS chain, in response to environmental parameters and during transition of bacteria from the free-living into intracellular state [10,50]. This indicates great plasticity of LPS synthesis and a vast role of methyl groups in adaptation to environmental changes. Perhaps, different perception of the osmotic pressure by the *oatB* mutant, due to the lower content of O-PS, forces similar changes. Other features of the LPS molecule affecting the hydrophobicity value (the length of O-PS and the structure of lipid A) can be omitted since they are the same for all studied strains. 

Recently, the O antigen has been shown to be a crucial determinant governing the recognition and uptake of different strains of *Escherichia coli* by *A. castellanii*, which makes it a potential anti-predator defense molecule. On the other hand, the presence of an LPS O-antigen chain was not required for the internalization of the bacteria by these amoebae [51]. Additionally, March et al. [52] showed that the LPS *O*-polysaccharide of *Klebsiella* contributed to its resistance to predation by the social amoeba *Dictyostelium discoideum*. In the present study, co-culture assays were carried out in order to establish the influence of the depletion of OatB on *M. loti*–*A. castellanii* interactions. Since the amoebae internalized both the *OatB*-depleted mutant and its parental strain with the same efficiency (Figure 5A)—although they did so at different rates—either the presence of O chains did not matter in the uptake of bacteria, or their diminished quantity was sufficient for the process. In the course of the experiments, we observed that the *oatB* mutant was internalized faster by amoebae than the WT strain. However, because the values of hydrophobicity determined for both strains were statistically insignificant, this could not have been the cause of the different rate of bacterial internalization. Moreover, Matz and Jürgens found that hydrophobic and electrostatic cell surface properties of bacteria did not severely affect feeding rates of heterotrophic nanoflagellates [53]. Thus, the cause of this effect might be the different participation of mannose-dependent lectins in the process in both strains.

However, we determined that mutation in *oatB* had an impact on the viability of bacteria internalized by *A. castellanii*. This could be directly observed in the LIVE/DEAD assay (BacLight kit) or inferred based on the amount of bacterial CFU released from the amoebae (Figure 5). The liberation of multilamellar bodies (MLBs) (structures which require for their production the presence of digestible bacteria) from amoebae co-cultured with *oatB* cells into the extracellular milieu provided additional evidence for this finding (Figure 6F) [54].

As a response to intruders, activated phagocytic cells, including amoeba cells, express inducible nitric oxide synthase, an enzyme responsible for the production of nitric oxide, which is an essential mediator of defense. In our study, the expression of iNOS was stimulated exclusively when amoebae were activated with live *oatB* mutant cells (Figure 7A), but not the WT strain or S-LPSs derived from the two strains. The fact that equal amounts of S-LPSs, differing in the level of *O*-acetyl substitution of O-PS, did not induce NO^2−^ production by the amoebae, may imply that the mechanism protecting the bacteria against nitrosative stress is not associated with the structure of O antigen chains, but their abundance. Similar results were obtained by Bagüés and colleagues for macrophages activated by smooth and rough *Brucella* spp. Those authors hypothesized that it was not the O-PS moieties that triggered the defense response but the ligands that are normally covered by them [55]. 

To clear invasion of microorganisms, phagocytes are also supposed to generate reactive oxygen species. However, this conclusion was obtained from studies focused on ROS-dependent damage to bacteria by macrophages in the cytoplasm, while the mechanisms by which ROS damage bacteria in phagosomes are unclear [56]. Our study revealed that, the production of phagocytic ROS by amoebae activated with whole cells of bacteria was higher for the WT strain than for the *oatB* mutant at 2-, 4-, and 24-h co-culture assays. At the same time, the titers of WT released from amoebae were significantly higher in comparison to the *oatB* mutant. Similar observations were made by Halablab et al. [32] for virulent *Legionella pneumophila* in comparison with an avirulent strain. The virulent strain, being able not only to survive inside *Acanthamoeba* but also to proliferate, generated higher formazan amounts inside phagosomes (indicating more intense respiratory burst) than the avirulent strain. Since, O_2_^−^ (whose level was estimated by us), as a charged anion, cannot cross membranes, the primary targets of phagocytic O_2_^−^ must be extracytoplasmic or after dismutation to H_2_O_2_ it can diffuse the phagosomal membrane to destroy bacteria [56]. However, in the sensitivity assay with use of H_2_O_2_, the percentage of recovered cells of the *oatB* mutant at the end of experiment was the same as for the WT strain, compared to the initial titers. This could be due to the higher expression of catalases and/or peroxidases, i.e., enzymes which provide H_2_O_2_-detoxifying activity, by the *oatB* mutant, thus compensating for the lower content of O antigen particles in its OM [57]. All experiments collectively might indicate that, ROS favors the intracellular route of the WT strain rather than it has a bactericidal effect. This phenomenon has been explained for some intracellular pathogens, and the ROS production was indicated to favor the infection of host cells by influencing e.g., iron availability for pathogens and inactivation of some cysteine proteases, thus providing protection against phagosomal proteolysis [58]. However, the data from our study are not sufficiently clear to explain the ROS role in *M. loti*-*A. castellanii* interactions. The only conclusion that can be drawn is that, ROS production inside phagosomal compartments seems to depend on the structure of LPS because its equal amounts are liberated by amoebae during phagocytosis (at the given p.i. times) upon stimulation with whole cells or LPS (of both the WT strain and the *oatB* mutant; Figure 7B).

Collectively, these results demonstrate that disruption of the integrity of the *oatB* gene of *M. loti* alters the physicochemical properties of its cell wall and impairs its survival within *A. castellanii* by making it vulnerable to nitrosative stress and/or the action of cationic peptides and/or lytic enzymes.

## 4. Materials and Methods

### 4.1. Strains and Culture Conditions

The bacterial strains and plasmids used in this work are listed in Table 2. *Mesorhizobium loti* strains were grown in a liquid 79CA medium [59] on a rotary shaker (160 rev/min) at 28 °C for 24 (WT, NZP2213.1(pBGoatB)) and 48 h (NZP2213.1); *Escherichia coli* was grown in a lysogeny broth (LB) medium at 37 °C [60]. Antibiotics, when needed, were used at the following final concentrations (µg·mL^−1^): kanamycin, 40 for *E. coli*, 30 for *Mesorhizobium*; rifampin, 40; gentamicin 5 for *E. coli*, 12.5 for *Mesorhizobium*.

The Neff strain *Acanthamoeba castellanii* (ATCC 30010) was grown in 300 mL conical flasks in a PYG (Peptone-Yeast-Glucose) medium with shaking as described previously [61]. A Bürker chamber was used to estimate the density of amoebae in the culture.

### 4.2. Construction of Plasmid for Complementation

The plasmids used in this work are listed in Table 2. Standard protocols for genomic DNA isolation, PCR, molecular cloning, transformation, and DNA analysis were used [60]. The pBGoatB plasmid used for the complementation trial was constructed by cloning the PCR product covering the 3′-end of the *oatA* gene, the *oatA-oatB* intergenic region and the *oatB* gene with downstream sequences indispensable for transcription termination, into the SmaI site of the pBBR1MCS-5 vector [64]. For that purpose, the genomic fragment was amplified with Pfu polymerase (Thermo Fisher Scientific, Waltham, MA, USA) using the *oatB*complFW: 5′-CTTTCGCGTTCTCAGTCGTGTTC-3′ and the *oatB*compRV: 5′-GTAATCTCCTTTCTAGCATATCG-3′ primers. The primers were designed based on the genomic sequence of the *Mesorhizobium ciceri* bv. *biserrulae* WSM1284 strain. The sequence of the insert was deposited in GenBank under accession number MH626640. The pBGoatB plasmid was transferred from *E. coli* S17-1 to the NZP2213.1 mutant by conjugation, and gentamycin-resistant clones were checked for the presence of the desired plasmid. Bacterial mating experiments were performed as described by Simon et al. [62]. DNA sequencing of the plasmid construct was performed in Genomed (Warsaw, Poland). Sequence data were analyzed with DNASTAR-Lasergene software (DNASTAR Inc., Madison, WI, USA). Putative homologs of OatB were identified using BLASTp [65].

### 4.3. Lipopolysaccharide Extraction and Purification and the Sugar Composition of LPS and O-PS

LPS was extracted from bacterial cells of the complemented NZP2213.1(pBGoatB) strain by the hot phenol-water method [66] as described previously [7]. LPS preparations were obtained from both phases and purified by repeated ultracentrifugation at 105,000× *g* for 4 h. *O*-polysaccharide portion was obtained only from the phenol-soluble S-LPS by mild acid hydrolysis (1.5% acetic acid at 100 °C for 3 h). After removal of lipid A by centrifugation, the supernatant containing O-PS was fractionated by GPC on a Sephadex G50 fine column (1.8 × 80 cm) and eluted in a void volume. Similarly, O-PSs of the WT and mutant NZP2213.1 strains were obtained from LPS extractable from the phenol phase as previously described [7,20]. 

For analysis of neutral and amino sugar, such as alditol and amino alditol acetates, the polysaccharides (O-PSs and degraded PSs) were hydrolyzed with 2 M CF_3_CO_2_H (120 °C, 2 h), *N*-acetylated, and reduced with NaBD_4_; this was followed by acetylation with a 1:1 (*v*/*v*) mixture of acetic anhydride and pyridine (85 °C, 0.5 h). To release acidic monosaccharides, polysaccharides (dgPS) were subjected to methanolysis (1 M methanolic-HCl, 85 °C, 16 h), carboxyl-reduced with NaBD_4_ in aqueous 50% methanol, hydrolyzed with 2 M CF_3_CO_2_H (120 °C, 2 h), reduced with NaBD_4_, and acetylated as above. Monosaccharides such as alditol and amino alditol acetates were identified by GLC-MS on a Agilent Technologies instrument 7890A (Agilent Technologies, Wilmington, DE, USA) connected to an MSD (inert XL EI/CI, Agilent Technologies) detector. The chromatograph was equipped with a capillary column (HP-5MS, 30 m × 0.25 mm, Agilent Technologies) applying a temperature gradient of 150 °C (5 min) to 310 °C at 5 °C min^−1^.

### 4.4. Hydrophobicity Test—Microbial Adhesion to Hydrocarbons (MATH)

The hydrophobicity of the bacteria was tested using the MATH method as described previously [67]. Bacteria obtained from the plate cultures were suspended in PUM buffer (22.2 g K_2_HPO_4_ × H_2_O, 7.26 g KH_2_PO_4_, 1.8 g urea, 0.2 g MgSO_4_ × 7H_2_O, 1 L MilliQ water) to an optical density of about 0.5 (OD_1_) at 405 nm. 200 µL-aliquots of bacterial suspensions were supplemented with 100 µL of dodecane, left for 10 min at room temperature, and vortexed exhaustively for 120 s. After 15-min equilibration, the optical density of the lower phase was measured (OD_a_) on a microplate reader (Biochrom Asys UVM 340). The degree of hydrophobicity was calculated as follows: % hydrophobicity = 100 − 100(OD_a_/OD_1_).

### 4.5. 1-N-Phenyl-1-Naphthylamine Uptake Assay

Outer membrane permeability of the WT, NZP2213.1, and NZP2213.1(pBGoatB) strains was determined using the *N*-phenyl-1-naphthylamine uptake assay [67] with a minor modification. Overnight broth cultures of *log*-phase bacteria were adjusted to an optical density (OD_600_) of 0.2, harvested, and diluted in an equal volume of 5 mM HEPES (4-(2-hydroxyethyl)-1-piperazineethanesulfonic acid) buffer (pH 7.2). One-hundred-microliter portions of the bacterial suspensions were mixed with 95 µL of the HEPES buffer and 5 µL of a 0.5 mM NPN solution in acetone. Two controls were performed for each analyzed strain; a first contained 100 µL of the HEPES buffer and 100 µL of the bacterial suspension, whereas a second contained 95 µL of the HEPES buffer and 5 µL of the NPN solution. The intensity of fluorescence was measured in the Tecan Infinite 200PRO microplate reader (Tecan Austria GmbH, Gröding, Austria) for 18 min at 2-min intervals using excitation and emission wavelengths of 355 and 405 nm, respectively. To standardize the data, viable cells from the bacterial suspensions were plate counted. Data are reported as relative fluorescent units (RFU) per CFU.

### 4.6. Sensitivity of Bacteria to Membrane Disruption Agents, Acidic pH, and Hydrogen Peroxide

Overnight *M. loti* cultures grown on 79CA medium were adjusted to an OD_600_ of 0.4, and appropriate amounts of cells were washed once in normal saline followed by dilution in a fresh portion of saline. For the filter disc assays, 100-µL aliquots of cells were mixed with 4 mL of 79CA soft agar (6 g/L) and poured onto plates (20 mL) with 79CA medium. After 30 min, 6-mm diameter sterile paper disks were applied to the center of the plates and then spotted with 5 µL of the following test agents: aqueous solutions of sodium dodecyl sulfate (SDS)—100 g/L, crystal violet (CV)—4 mg/mL, or polymyxin B (PmB)—4 mg/mL. The sensitivity of the bacteria to sodium deoxycholate (DOC) and ethanol (EtOH) was evaluated using round gradient plates according to a previously described method [68]. The contents of 100-mL 79CA gradient plates (50 mL per layer) were poured into large, round (13.5 cm in length) Petri dishes. The selective top layer contained either 24 mM DOC or 8% (*v*/*v*) ethanol. In one version of the ethanol experiments, CaCl_2_ (2.5 mM) and MgSO_4_ (2.5 mM) were added to both layers (EtOH + MC). The cultures to be tested (30 µL aliquots) were streaked evenly across the plates and incubated for 72 h at 28 °C, followed by measurement of the diameter/length of growth inhibition zones.

The ability of the *M. loti* strains to grow at various pH values (4.5–7.0) was measured in broth cultures on 79CA medium as an increase in the optical density at 600 nm during 24 h (ΔOD_600_). 100 µL aliquots were used as an inoculum for the pH series. The viability of the strains after 24-h incubation was checked by plating on 79CA medium. An acidic environment was created using 2-(*N*-morpholino)ethanesulfonic acid (MES) [68]. The ΔOD_600_ values obtained for the particular pH levels were converted into percentage of ΔOD_600_ for cultures growing under normal conditions (100%).

The growth of *M. loti* in the presence of hydrogen peroxide was examined in broth cultures. 900-µL portions of pre-washed bacterial cells were exposed to 100 µL of H_2_O_2_ at a final concentration of 10 mM for 15 min with continuous agitation ensuring better distribution of the factor followed by triple wash in saline, and determination of CFU. The data are presented as percentage of CFU of untreated bacteria (100%).

### 4.7. Co-Culture Experiments

Co-culture experiments were carried out according to the Alsam et al. [39] method with minor modifications. Portions of amoebae containing 5 × 10^5^ cells were placed in 24-well tissue culture plates for 24 h at 28 °C to adhere. The obtained monolayers were washed three times with Page’s amoeba saline (PAS; pH 7.4) and finally re-suspended in 1 mL of PAS. The turbidity of the *M. loti* cultures was adjusted to the McFarland standard of 1, and 1-mL-portions were harvested, washed three times with PAS, and suspended in an equal portion of fresh PAS prior to co-incubation with *A. castellanii*. The amoeba monolayers were then exposed to 40 µL of washed bacteria, resulting in a multiplicity of infection (MOI) of approximately 10 bacteria per amoeba. Infection was synchronized by centrifugation at 500× *g* for 10 min. 

In the invasion assay, *M. loti*-challenged amoebae were allowed to internalize the bacteria for 4 and 24 h at 28 °C. Following the co-incubation, the extracellular medium was aspirated, and the amoeba layer was washed three times with PAS. The remaining extracellular bacteria were killed with streptomycin and ampicillin (at a concentration of 200 µg·mL^−1^) for 4 h, which was followed by triple washing with PAS. The amoebae from the invasion assay were used for: (i) the LIVE/DEAD study; (ii) TEM preparation; and (iii) CFU determination of internalized bacteria. In counting experiments, the intracellular bacteria were first liberated from amoebae by sonication in an ultrasonic bath (at 20–40 kHz, for 10 min) and then passed ten times through a 26-gauge needle, after which the titers of liberated bacteria were determined by serial dilutions and plating on 79CA plates.

For the attachment inhibition assay, the amoebae were exposed to solutions of saccharides (100 mM·L^−1^ glucose, galactose, rhamnose, and mannose in phosphate buffer saline (PBS) at pH 7.4) prior to co-cultivation with *M. loti* strains as described earlier [8]; the rest of the experiment was carried out as in the invasion assay, except that the co-culture was carried out for 1 h, and no antibiotics were used after the final wash. The CFUs were expressed as percentage of CFUs of bacteria liberated from untreated amoebae (100%).

In the phagolysosome fusion inhibition assay, the amoeba monolayers were pre-treated prior co-culture with 20 or 40 mM NH_4_Cl in PBS for 1 h at 28 °C. Then, the experiment was performed according to the procedure described for the invasion assay with 4-h co-incubation, and the titers of intracellular bacteria were obtained as before.

### 4.8. LIVE/DEAD Assay

To determine the viability of internalized *M. loti*, we employed the method described by Steinert et al. [69]. For LIVE/DEAD staining, 4-h and 24-h invasion assays were performed as described above. Images were collected on a Zeiss LSM780 laser scanning confocal microscope using 488 nm excitation light from an Argon laser for SYTO9 and 561 nm excitation light from a DPSS laser for Propidium Iodide (PI). Thus, damaged cells were identified by red fluorescence, while metabolically active cells were identified by green fluorescence. The images were used to calculate the percentage of *A. castellanii* population infected with the bacteria and the percentage of metabolically active and inactive intracellular bacteria, as well as to assess the number of bacteria (total, LIVE, DEAD) per single amoeba cell. All assessments were based on more than 200 randomly selected amoebae cells, collected from at least five fields. To estimate the total number of bacteria internalized over the given incubation period, the titers of amoeba were evaluated at the end of the experiment using the Bürker chamber.

N_LIVE/DEAD_ = log (n × A*c*_t_)

N—log number of LIVE or DEAD bacteria;

n—average number of LIVE (or DEAD) bacteria per one amoeba;

A*c*_t_—the amount of amoeba at the end of co-culture assay.

### 4.9. Nitrosative and Oxidative Stress Assays 

The efficiency of nitric oxide radicals (NO^**∙**^) production was estimated as the amount of NO_2_^−^ in the culture medium, using the method described by Inoue et al. [70] with some modifications. Amoeba cells were activated with pre-washed bacteria at a MOI of 10 or with a lipophilic fraction of LPSs (final concentration 1 µg·mL^−1^) obtained according to a method described above in the Section 4.3. The experiment was run as described for the invasive assay, except that the final volume of the reaction mixture was 250 µL. After the amoebae had been exposed to the respective factors for 2, 4, 6, and 24 h at 28 °C, the accumulation of nitrite (the stable product of nitric oxide breakdown) in supernatants was measured using the Griess reaction according to Koo et al. with a minor modification [31]. Briefly, 100 µL-aliquots of cell-free culture supernatants from each well were placed in duplicate in a 96-well microplate and incubated for 5 min with an equal amount of 1% sulfanilamide in 5% phosphoric acid and then with 100 µL of 0.1% *N*-(1-naphtyl)ethylendiamine dichloride. Formation of diazonium was monitored at 550 nm in a microplate reader (Asys UVM 340, Asys Hitech GmbH), and the concentration of NO in each sample was calculated (µmol per 5 × 10^5^ amoeba cells) in relation to a standard calibration curve constructed for NaNO_2_. The final results were expressed as percentage of NO produced by non-treated amoebae (100%).

Superoxide anion production (O_2_^−^) by NADPH oxidase in bacteria- or LPS-activated amoebae was measured using Halablab et al.’s [32] method with minor modifications. Briefly, portions of amoebae containing 5 × 10^6^ cells each were placed in 24-well tissue culture plates and left for 24 h at 28 °C to adhere. This step was followed by disposable wash with PAS. Then, 0.5 mL of PAS was added to all wells. Control wells (blank) were additionally supplemented with an equal amount of 10 mM iodoacetamide to inhibit phagocytosis. After 2-h incubation at 28 °C, the supernatant was carefully removed from all wells. Next, the wells containing (iodoacetamide-treated and control) amoebae were supplemented with either pre-washed bacteria at 5 × 10^7^ (MOI 10) (bacteria-induced activation) or 10-µL portions of stock solution (25 µg·mL^−1^) (LPS-induced activation). Finally, to each well was added amoeba saline to a final volume of 160 and 80 µL of freshly prepared nitro blue tetrazolium at 1 mg·mL^−1^. Infection was synchronized by centrifugation of plates at 500× *g* for 10 min, and incubation was carried out for 2, 4, 6, and 24 h at 28 °C. After the incubation, amoeba cells were washed twice in warm PBS and once with methanol to completely remove non-reduced-NBT, after which they were air dried. The NBT, reduced to formazan particles, deposited inside the amoebae was then dissolved by adding 120 µL of 2 M KOH and 140 µL of DMSO with gentle shaking for 10 min in room temperature [71]. The dissolved formazan solution was then transferred to a 96-well plate, and A_620_ was read on a microplate reader (Tecan Infinite 200PRO, Tecan Austria GmbH).

### 4.10. The Effect of Amoeba Lysate and Antibiotics Treatment on Bacterial Cell Morphology

To prepare crude amoeba homogenate, *A. castellanii* cells were harvested, washed twice in PBS, suspended in a fresh portion of PBS to obtain a cell density of approximately 7 × 10^7^ cell/mL, and then disintegrated using a Potter tissue grinder held in an ice bath. Remnants of whole cells and nuclei were removed at 4 °C by centrifugation at 500× *g* for 5 min; then mitochondria were pelleted at 10,000× *g* for 10 min. The protein content in the homogenate was determined by the Bradford method. The homogenate was immediately used for *M. loti* treatment. 

100-µL portions of WT and NZP2213.1, obtained as for the sensitivity assays, were suspended, after a final wash, in 100 µL of an appropriate buffer (PBS pH 7.3 or 100 mM acetate buffer pH 5.0) and treated with an equal volume of the crude amoeba homogenate (containing 1.5 mg/mL of total proteins). In control samples, the saline solution was added instead of the amoeba lysate. The samples were incubated for 24 h at 28 °C, followed by a double wash in PBS.

For drug treatments, aqueous stock solutions of cephalexin and mitomycin C were prepared at concentrations of 1 and 0.5 mg/mL, respectively. Saline-washed bacteria (OD_600_ at 0.4) were treated for 24 h at 28 °C with cephalexin (3 µg/mL) and mitomycin C (200 ng/mL). The bacterial preparations were examined by contrast phase microscopy and scanning electron microscopy (SEM).

### 4.11. ^1^H NMR Spectroscopy

For the ^1^H NMR experiment, the O-PS samples were deuterium-exchanged by freeze-drying from D_2_O and then examined in 99.98% D_2_O using acetone (δ_H_ 2.225) as an internal or external standard. ^1^H NMR spectra were recorded on a Varian Unity plus 500 MHz instrument with standard Varian software (the O-PSs of the WT and mutant NZP2213.1 strains) or a Bruker 500 MHz instrument (Ascend 500) with standard Bruker software (complemented NZP2213.1 O-PS).

### 4.12. Atomic Force Microscopy (AFM)

To study the structural features of individual bacterial cells, *M. loti* samples were prepared for AFM according to a method described by Zdybicka-Barabas et al. [72] with slight modifications. Briefly, overnight cultures of the bacteria were diluted in a fresh portion of 79CA medium to OD_600_ = 0.2. Then, 100-μL portions of the cultures were centrifuged at 8000× *g* for 10 min at 4 °C. To remove exopolysaccharide, bacterial pellets were washed once with 0.5 M NaCl, then twice with normal saline, and again twice with 200 µL-portions of apyrogenic water. After a final centrifugation, the bacteria were suspended in 50 µL of water, and 5 µL-portions of the bacterial solution (total number of cells ~5 × 10^5^) were deposited on bare mica discs and allowed to dry overnight at room temperature. The surfaces of the bacterial cells were imaged using a NanoScope V AFM (Veeco, Oyster Bay, NY, USA) (Analytical Laboratory, Faculty of Chemistry, Maria Curie-Sklodowska University, Lublin, Poland). All measurements, with the exception of the DMT modulus (5 N·m^−1^ TAP150A, Bruker, Billerica, MA, USA), were made in the “Peak Force QNM” operation mode using a silicon tip with a spring constant of 20 N·m^−1^ (NSG30, NT-MDT, Moscow, Russia). The following parameters were analyzed: (i) height and peak force errors, showing the topography of the examined strains; (ii) DMT (Derjaguin, Muller and Toporov) modulus, adhesion, and deformation, reflecting bacterial cell surface stiffness; and (iii) adhesion forces between the cell surface and the tip. The data were analyzed with Nanoscope Analysis software version 1.40 (Veeco, Oyster Bay, NY, USA). Roughness was measured on 500 × 500 nm images of the bacterial cell surface over surface areas of 150 × 150 nm. The root-mean-square surface roughness of the cells was calculated from at least 30 fields collected in total from three bacterial cells coming from different cultures.

### 4.13. Electron Microscopy

For transmission electron microscopy, amoeba trophozoites infected with *M. loti* in the 24-h invasive assay were harvested and prefixed for 2 h at 4 °C in 2.5% glutaraldehyde in 0.1 M cacodylate buffer (pH 7.3) with 3% of sucrose and 2.5 mM CaCl_2_. The samples were then washed twice in 0.1 M cacodylate buffer, post-fixed in 1% osmium tetroxide for 1 h at 4 °C, stained with 1% aqueous uranyl acetate, and dehydrated with an ascending ethanol series. The resulting pellets were then infiltrated with and embedded in LR White resin. After polymerization (50 °C for 2 days), the resin blocks were sectioned, post-stained with uranyl acetate and lead citrate, and examined under a LEO 912AB (Zeiss, Oberkochen, Germany) energy filter transmission electron microscope.

Scanning electron microscopy was used to visualize the effect of the amoeba lysate and the investigated antibiotics on the morphology of *M. loti* cells. After fixation with 4% glutaraldehyde in a 0.1 M phosphate buffer, pH 7.0, the mesorhizobial cells were treated with OsO_4_, dehydrated stepwise in a graded acetone series, dried, and sputter-coated with gold using a K550X sputter coater (Quorum Technologies, Laughton, UK). Cell morphology was analyzed using a Vega 3 scanning electron microscope (Tescan, Brno-Kohoutovice, Czech Republic).

### 4.14. Statistical Analysis

All experiments were performed independently three times in duplicate. The results were analyzed statistically with GraphPad Prism 6.0 software and presented as means ± standard deviation (SD) of three independent experiments. The significance level was established using two-tailed Student’s *t*-tests.

## 5. Conclusions

It has been shown that bacterial inoculants developed in laboratories and introduced into soil show inconsistent field performance. The low effectiveness of some of these agricultural amendments is mainly due to the rapid decline of populations of active cells, caused, among others, by protozoan grazing. In our previous report, we showed that *M. loti* NZP2213 (WT) was able to resist *A. castellanii* grazing, and, as a result of interaction with the amoeba, developed larger numbers of nodules on the host plant. Thus, this strain was shown to be a good candidate for potential use as an inoculant of *L. corniculatus* [8]. Knirel et al. [45] showed that close proximity of bacteriophages leads to frequent spontaneous mutations, resulting in variations in O antigen biosynthesis and *O*-acetylation and altered phage sensitivity in *E. coli*. The data obtained in the present study indicate that the combined use of bacteria and *A. castellanii*, in the form of a consortium, as a plant inoculant may be more beneficial, since amoebae could support the persistence in the soil of the symbiotically effective wild-type strain, in a genetically unchanged form, by clearing symbiotically impaired strains, such as the *oatB* mutant, with changed and truncated LPS. Moreover, by shaping indigenous populations, amoebae could increase the competitiveness of *M. loti* NZP2213 in the rhizosphere.

## Figures and Tables

**Table 1:** Glycosyl composition of polysaccharide portions of *M. loti* WT, mutant NZP2213.1 and complemented NZP2213.1(pBGoatB) strains.

Glycosyl Residue	Molar Ratio ^a^		
	S-LPS from Phenol Phase		
	WT	NZP2213.1	NZP2213.1(pBGoatB)
2-*O*-methyl-6-deoxytalose	0.40	1.10	0.93
6-deoxytalose	15.00	7.5	13.4
Rhamnose	1.10	1.20	1.4
*N*-acetylquinovosamine	0.45	0.50	0.50
Glucose	1.30	1.20	2.0
Glucuronic acid	0.30	0.14	0.1
Galactose	1.00	0.70	0.16 ^c^
Galacturonic acid	0.10	0.10	ND ^c^
Glucosamine	1.00	1.00	1.0
Heptose	2.80	3.00	2.0
KDO ^b^	0.50	0.50	0.05 ^c^

ND—none detected; ^a^: Molar ratio normalized to glucosamine (1.00); ^b^: 3-deoxy-d-manno-2-octulosonic acid determined as the carboxyl-reduced alditol acetate, containing two deuterium atoms at C-1; ^c^: The reduced amount of sugars, which are the inner core compounds, probably indicates coprecipitation of lipid A and shorter core oligosaccharides after incomplete mild acid hydrolysis of lipopolysaccharide (LPS) and release of degraded PS.

**Table 2:** Bacterial strains and plasmids used in this study.

Bacterial Strains and Plasmids	Description	Reference or Source
***E. coli*** **strains**		
*S17.1*	294 derivative RP4-2-Tc::Mu-Km::Tn7 chromosomally integrated	[62]
*DH5α*	*supE44ΔlacU169*(ϕ80Δ*lacZ*Δ*M15*)*hsdR17recA1endA1gyrA96thi-1relA1*	[60]
***M. loti*** **strains**		
NZP2213(HAMBI1129)	Wild-type Rif ^R^, Nod ^+^ Fix ^+^	[63]
NZP2213.1	NZP2213::Tn5Rif ^R^, Km ^R^, Nod^+^Fix ^−^	[7]
NZP2213.1(pBGoatB)	Carrying pBGoatB complementation plasmid	This work
**Plasmids**		
pBBR1MCS-5	mob, Gm ^R^	[64]
pBGoatB	pBBR1MCS-5 with 2005-bp fragment carrying 3′-end of *oatA* gene, *PoatB*, and the whole *oatB* gene, cloned into SmaI in the direction corresponding to P*lac*	This work

## Supplementary Materials

Supplementary materials can be found at http://www.mdpi.com/1422-0067/19/11/3510/s1. Figure S1. Phenotypic effect of the *oatB* mutation in *Mesorhizobium loti* revealed by: (A) SDS-PAGE and, (B) immunoblot analysis. Phenol-soluble S-LPS samples were separated in 12.5% SDS–Tricine polyacrylamide electrophoresis gel [73], and bands were visualized by silver staining after oxidation with periodate according to the method of Tsai and Frasch [74]. For immunochemical analysis, LPSs were transferred to Immobilon P (Millipore). Rabbit antibodies against S-LPS from the phenol phase of *M. loti NZP2213* were raised according to the schedule described by Biosca et al*.* [75]. The hybridization step was conducted with 1:750 diluted primary antibody solution, which was detected using alkaline phosphatase-conjugated goat antirabbit antibodies (Sigma). Blots were developed with nitroblue tetrazolium and 5-bromo-4-chloro-3-indolylphosphate toluidine (Sigma) for 5 to 15 min. Lines: 1. S-LPS of *NZP2213* (WT) (4 µg); 2. S-LPS of the NZP2213.1 mutant (3 µg); 3. S-LPS of the *NZP2213* (WT) (3 µg); 4. S-LPS of the NZP2213.1(pBGoatB) complemented strai (3 µg).

## Abbreviations

AFM	atomic force microscopy
Cef	cephalexin
DOC	sodium deoxycholate
EtOH	ethanol
iNOS	inducible nitric oxide synthase
LPS	lipopolysaccharide
MCVs	*Mesorhizobium*-containing vacuoles
MES	(*N*-morpholino)ethanesulfonic acid
MitC	mitomycin C
MLBs	multilamellar bodies
MOI	multiplicity of infection
NBT	nitro blue tetrazolium
NMR	nuclear magnetic resonance
NO	nitric oxide
iNOS	inducible nitric oxide synthase
NOX	NADPH oxidase
NPN	1-*N*-phenylnaphthylamine
OM	outer membrane
O-PS	O-antigen, O-polysaccharide chain
PAS	Page’s amoeba saline
PBS	phosphate buffer saline
PGPR	plant growth promoting bacteria
PI	propidium iodide
PmB	polymyxin B
RFU	relative fluorescent units
RMS	root-mean-square roughness
RNS	reactive nitrogen species
ROS	reactive oxigen species
SDS	sodium dodecyl sulfate
SEM	scanning electron microscopy
TEM	transmission electron microscopy
TLRs	Toll-like receptors
