# Supplementary material for: A Mutation in the Mesorhizobium loti oatB Gene Alters the Physicochemical Properties of the Bacterial Cell Wall and Reduces Survival inside Acanthamoeba castellanii
Source: Int J Mol Sci. 2018 Nov 8;19(11):3510. doi: 10.3390/ijms19113510 (PMC6274867; doi:10.3390/ijms19113510)
Supplement: Supplementary file 1 [file ijms-19-03510-s001.zip › ijms-368063 suppl Figure S1 for proof.docx]

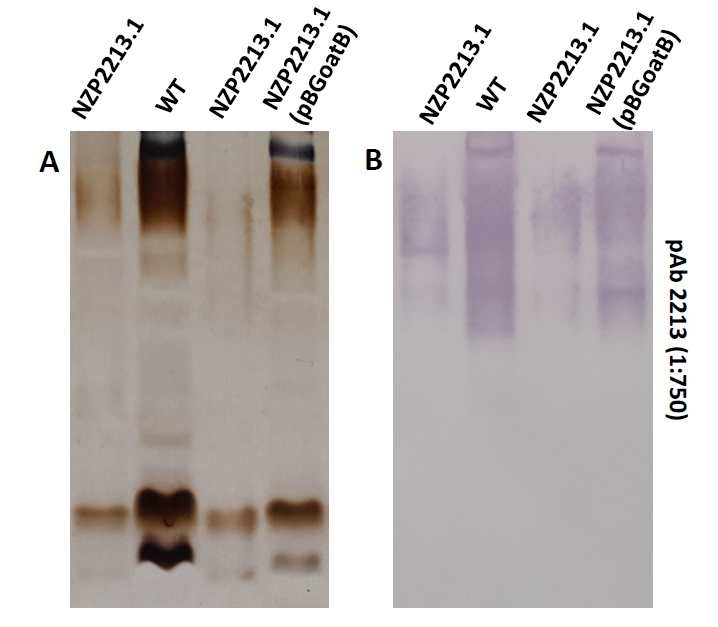


**Figure S1.** Phenotypic effect of the *oatB* mutation in *Mesorhizobium loti* revealed by: (**A**) SDS-PAGE and, (**B**) immunoblot analysis. Phenol-soluble S-LPS samples were separated in 12.5% SDS–Tricine polyacrylamide electrophoresis gel [73], and bands were visualized by silver staining after oxidation with periodate according to the method of Tsai and Frasch [74]. For immunochemical analysis, LPSs were transferred to Immobilon P (Millipore). Rabbit antibodies against S-LPS from the phenol phase of *M. loti* *NZP2213* were raised according to the schedule described by Biosca et al*.* [75]. The hybridization step was conducted with 1:750 diluted primary antibody solution, which was detected using alkaline phosphatase-conjugated goat antirabbit antibodies (Sigma). Blots were developed with nitroblue tetrazolium and 5-bromo-4-chloro-3-indolylphosphate toluidine (Sigma) for 5 to 15 min. Lines: 1. S-LPS of *NZP2213* (*WT*) (4 µg); 2. S-LPS of the *NZP2213.1* mutant (3 µg); 3. S-LPS of the *NZP2213* (*WT*) (3 µg); 4. S-LPS of the *NZP2213.1*(pBGoatB) complemented strai (3 µg).
